# Human-Centric Spatial Cognition Detecting System Based on Drivers’ Electroencephalogram Signals for Autonomous Driving

**DOI:** 10.3390/s25020397

**Published:** 2025-01-10

**Authors:** Yu Cao, Bo Zhang, Xiaohui Hou, Minggang Gan, Wei Wu

**Affiliations:** 1 School of Automation, Beijing Institute of Technology, Beijing 100081, China; 3220231086@bit.edu.cn (Y.C.); zbpt516@163.com (B.Z.); agan@bit.edu.cn (M.G.); wuwei@bit.edu.cn (W.W.); 2 National Key Lab of Autonomous Intelligent Unmanned Systems, Beijing Institute of Technology, Beijing 100081, China

**Keywords:** electroencephalogram (EEG), automatic driving, spatial cognition, human–machine cooperation

## Abstract

Existing autonomous driving systems face challenges in accurately capturing drivers’ cognitive states, often resulting in decisions misaligned with drivers’ intentions. To address this limitation, this study introduces a pioneering human-centric spatial cognition detecting system based on drivers’ electroencephalogram (EEG) signals. Unlike conventional EEG-based systems that focus on intention recognition or hazard perception, the proposed system can further extract drivers’ spatial cognition across two dimensions: relative distance and relative orientation. It consists of two components: EEG signal preprocessing and spatial cognition decoding, enabling the autonomous driving system to make more contextually aligned decisions regarding the targets drivers focus on. To enhance the detection accuracy of drivers’ spatial cognition, we designed a novel EEG signal decoding method called a Dual-Time-Feature Network (DTFNet). This approach integrates coarse-grained and fine-grained temporal features of EEG signals across different scales and incorporates a Squeeze-and-Excitation module to evaluate the importance of electrodes. The DTFNet outperforms existing methods, achieving 65.67% and 50.65% accuracy in three-class tasks and 84.46% and 70.50% in binary tasks. Furthermore, we investigated the temporal dynamics of drivers’ spatial cognition and observed that drivers’ perception of relative distance occurs slightly later than their perception of relative orientation, providing valuable insights into the temporal aspects of cognitive processing.

## 1. Introduction

With the rapid development of autonomous driving technology, its application has become feasible in relatively simple environments. However, there are still many problems with autonomous driving technology, and it is still a long way from being fully applied [1,2,3,4,5]. For instance, current autonomous driving systems are highly susceptible to adverse weather conditions [1,2]. Moreover, when confronted with unexpected obstacles or emergencies on the road, these systems often struggle to make accurate judgments and respond promptly [3]. Failures in software or hardware components can further lead to severe safety risks [4]. These technical limitations necessitate human intervention in current autonomous driving systems [5]. In addition to safety challenges, existing autonomous driving technologies frequently fall short in ensuring driver comfort, particularly in achieving “human-like” driving behaviors. This disconnect can result in discrepancies between system decisions and driver expectations, leading to discomfort, anxiety, or even traffic accidents [6].

To address these challenges, a promising solution involves developing human-centric assistant driving systems based on electroencephalogram (EEG) signals [7]. By introducing the cognition of drivers into the driving system, it is possible to enhance both the safety and comfort of autonomous driving [8,9]. EEG, as a commonly utilized physiological signal, captures valuable cognitive information about drivers. It reflects their perception and understanding of the environment, offering insights into processes such as perception, judgment, and decision-making. As a reliable source of reference for autonomous driving systems, EEG data can effectively compensate for the limitations of onboard sensors [10].

Several studies about the EEG-based human-centric assistant driving system have been published in recent years [11,12,13,14,15,16,17,18,19,20,21,22,23,24,25,26], and most of them focus on drivers’ intention detection and hazard perception. Haufe et al. achieved a prediction 130 ms before the actual braking event using a linear discriminant analysis (LDA) classifier [11]. Zhang et al. proposed a model that includes hypergraph convolution for feature extraction, which outperforms the baseline in detecting various types of driving intentions, with an accuracy rate of 74.40% [13]. Teng et al. proposed an LDA model based on spatial-frequency features of EEG for detecting emergency braking intentions, achieving an accuracy of up to 94% [16]. As for driver’s hazard perception, a hazard perception classifier based on EEG signals was developed for the scenario where pedestrians cross the road [19]. The low-frequency activities in EEG signals were found to increase when a vehicle cut in, which could be used to predict the cutting-in behavior of other vehicles [21]. Zhang et al. proposed an improved neural network intersection collision prediction model based on EEG indicators and driving behaviors [24].

However, focusing only on these two aspects is far from enough to help the driving system make effective decisions and improve ride comfort. With the development of relevant research, researchers found that hazards occurring in different locations can also trigger different neural responses. Li et al. found that hazards in the peripheral visual area can induce larger amplitude EEG signal components than those in the central visual area [27]. Wang et al. found that the brain activities triggered by lateral risks are stronger than those triggered by longitudinal risks using fNIRS [28]. These studies indicate that the spatial cognition of drivers can be reflected in neuroimaging techniques.

This study established a human-centric spatial cognition detecting system based on drivers’ electroencephalogram signals for autonomous driving. By decoding the drivers’ spatial cognition, the driving system can set a more comfortable following distance and keep the vehicle within the safe range perceived by the drivers based on the position of the target that the driver is paying attention to. And it is no longer necessary to decode the drivers’ intention and hazard perception. However, there is no current research on spatial cognition detecting in driving scenarios based on EEG signals. Most of the EEG-based spatial cognition studies are based on simple scenes [29,30,31,32]. Kastrati et al. proposed EEGEyeNet, which is an EEG signal dataset for decoding gaze positions; by establishing the mapping relationship between EEG signals and annotated positions, it is possible to initially detect human spatial cognition [29]. Himmelberg et al. discovered that EEG signals can be utilized to decode the 3D movement direction of the observed target [31]. These studies have confirmed the feasibility of detecting spatial cognition from EEG signals.

To the best of our knowledge, this study is the first to explore and investigate the extraction of drivers’ spatial cognition based on EEG signals, addressing a critical gap in this research domain. We demonstrate the feasibility of detecting drivers’ spatial cognition, establishing a foundation for future advancements in this field. The key contributions of this study are as follows:

- **Pioneering a human-centric spatial cognition detecting system based on EEG signals:** This study introduces an EEG-based driver spatial cognition detecting system for the first time, which can equip the autonomous driving technology with high-level human spatial cognition to enhance its human likeness and comfort. The system can automatically preprocess EEG signals and decode drivers’ spatial cognition along two dimensions, namely relative orientation and relative distance, with two components: EEG signal preprocessing and spatial cognition decoding.
- **Proposing an innovative EEG decoding method called a Dual-Time-Feature Network:** We designed a novel Dual-Time-Feature Network (DTFNet), which employs a convolutional module and a gated recurrent unit (GRU) module to extract local and global temporal features from EEG signals, respectively. Furthermore, DTFNet incorporates a Squeeze-and-Excitation (SE) module to assess the importance of different electrodes, enhancing its ability to capture relevant spatial cognition features.
- **Comprehensive evaluation:** We conducted comparative experiments across different traffic environments with varying directions and distances, exploring the temporal dynamics of drivers’ spatial cognition. To rigorously validate the performance of the proposed method, we applied a five-fold cross-validation technique to evaluate its classification effectiveness against multiple baseline approaches.

The rest of this paper is structured as follows: Section 2 introduces the experimental setup and data process. Section 3 designs the methodology. Section 4 analyzes the experimental results. Section 5 draws the conclusion and discusses future research.

## 2. Experimental Procedure and Data Processing

**Figure 1:**
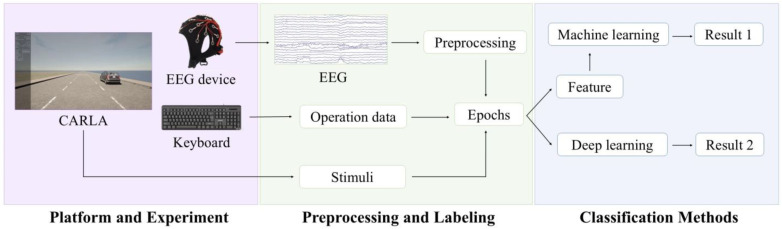
Flow diagram of experiment.

The complete experimental process is shown in Figure 1. Firstly, we recorded both EEG signals and operational data from participants while they engaged in the spatial cognition task within a simulated traffic environment. Next, the EEG signals were preprocessed and divided into epochs, with labels generated based on the operation data and the stimuli. Finally, we classified the EEG signals using both traditional machine learning models and deep learning models. For the traditional machine learning models, additional feature extraction was performed on the EEG signals prior to classification, whereas deep learning models classified the signals directly. Each procedure of the experimental process is detailed in the following content.

### 2.1. Experimental Platform and Experiment Design

#### 2.1.1. Experimental Platform

**Figure 2:**
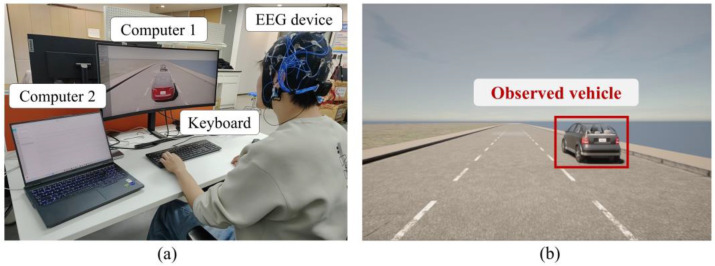
Driver-in-loop experimental platform. (**a**) Components of experimental platform and (**b**) driving simulation environment in CARLA.

As shown in Figure 2a, the experimental platform consists of two computers, an EEG acquisition device, and a keyboard. Computer 1 operates the driving simulation environment, where participants interact using a keyboard to record operational data. The participants’ EEG signals, which provide insights into their cognitive states, are collected via the EEG acquisition device. The EEG data are then transmitted to Computer 2 via Bluetooth for storage and further analysis. To ensure data synchronization between the two computers and the EEG device, we used the NTP (Network Time Protocol). For this study, we utilized the CARLA software (Version: Carla 0.9.15) [33] as the driving simulation environment and designed a circular three-lane map as the experimental setting, as shown in Figure 2b.

Our EEG acquisition device is the FLEX 2 Gel-32 Channel Wireless EEG Head Cap System by EMOTIV, which operates at a sampling frequency of 128 Hz and includes 32 acquisition channels. The accompanying software system incorporates a timestamp correction algorithm that, under optimal wireless connection conditions, reduces corrected timestamp errors to within ±0.5 ms, with a systemic error of approximately ±5 ms.

#### 2.1.2. Experimental Scene Design

We developed a spatial cognition experiment to collect and analyze participants’ EEG signals during the driving process. In the experiment, participants drove an autonomous vehicle from a first-person perspective while maintaining a speed of 30 km/h on the circular three-lane map for 10 min. The first-person driving perspective is depicted in Figure 2b. During the experiment, a randomly generated observed vehicle appeared on one of the three lanes ahead of the autonomous vehicle. Participants were instructed to record their spatial cognition of the observed vehicle using the keyboard.

The experiment focused on recording data only when the autonomous vehicle was traveling on straight sections of the map. Participants were allowed to rest while navigating the curved sections. Each observed vehicle was displayed for 2 s before disappearing, and the next vehicle was generated after a 0.1 s interval.

**Figure 3:**
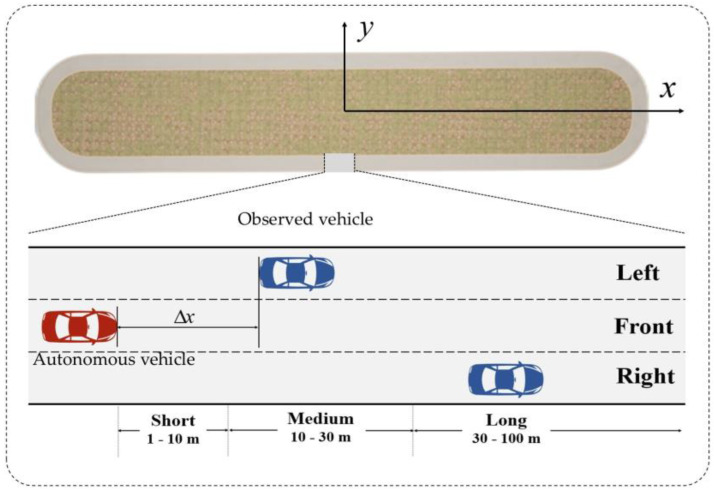
Experiment scene design in simulation environment.

As shown in Figure 3, a Cartesian coordinate system was established with the map’s center as the origin. The coordinates corresponding to the centerlines of the straight sections of the three lanes on the map are designated as $y_{0}$, $y_{1}$, and $y_{2}$, respectively. The position of the autonomous vehicle occupied by the participant is designated as $\left(x_{0},y_{1}\right)$, indicating that the vehicle is always driving in the middle lane. Let the relative distance between the generated observed vehicle and the autonomous vehicle be denoted as Δx, and let the lane be any of the three lanes. The position of the observed vehicle can be represented as $\left(x_{0}+\Delta x,y_{k},k\in \left\{1,2,3\right\}\right)$.

To balance the occurrence probabilities among the three relative distance groups—short, medium, and long—we defined these categories based on factors such as braking distance, safe following distance, and observation range during driving [34,35,36,37]. The relative distances were categorized as follows: short range (1–10 m), medium range (10–30 m), and long range (30–100 m). For each generated observed vehicle, one of these groups was randomly selected, and the specific relative distance (Δx) was drawn from a uniform distribution within the selected range.

In this paper, we decoded two spatial cognition dimensions, namely relative distance and relative orientation simultaneously. Considering that EEG signals contain significant noise and have a relatively low signal-to-noise ratio [38], we simplified the decoding tasks to reduce complexity. The relative distance decoding task was framed as a three-class classification problem—short, medium, and long distance. Similarly, the relative orientation decoding task was reduced to a three-class classification problem involving the left side, front, and right side.

#### 2.1.3. Participants

A total of 20 individuals participated in this study. The group consisted of 12 males and 8 females, aged 23 to 30 years. All participants had normal vision and hearing or corrected-to-normal vision and hearing, with no history of psychiatric or neurological disorders. Prior to the experiment, participants abstained from consuming any medications, tobacco, alcohol, or caffeine. Participation was entirely voluntary and conducted during regular working hours. Participants were informed that they had the right to withdraw from the study at any time without facing any penalties.

#### 2.1.4. Data Collection

Given that different participants possess varying levels of spatial cognition, we required participants to indicate their perception of the current relative distance after each observed vehicle was generated by pressing a key on the keyboard. Keys 1, 2, and 3 corresponded to perceptions of short, medium, and long distances, respectively. Additionally, we recorded the generation time, disappearance time, and the position coordinates of both the autonomous vehicle and the observed vehicle for each instance. On average, the number of valid data collected from each participant is 170.5 epochs.

### 2.2. Data Preprocessing and Annotation

The raw EEG data collected during the experiment contained substantial noise, necessitating preprocessing to enhance data quality [39,40]. Additionally, the EEG signals were segmented into epochs and annotated to facilitate subsequent analyses and related experiments.

#### 2.2.1. EEG Data Preprocessing

The preprocessing of EEG data in this study involved data selection, electrode localization, bandpass filtering to remove noise, re-referencing, and independent component analysis (ICA) for artifact removal.

**(1)**  **Data selection and electrode localization:** The data exported from our EEG device includes 116 channels, covering electrode connection quality, frequency data, and other metrics. For our analysis, we removed extraneous information and selected only the voltage data from 32 channels, with electrode positions determined according to the International 10–20 System.
**(2)**  **Band-pass filtering for noise removal:** A finite impulse response (FIR) filter was applied to perform bandpass filtering in the range of 0.5 to 40 Hz, reducing noise interference. For a given input time series, the output of an N-order finite impulse response filter was calculated in the following manner:
  (1) $$y\left(n\right)=\sum_{k=0}^{N-1}h\left(k\right)x\left(n-k\right)$$
  where $h\left(k\right)$ represents the filter coefficients.
**(3)**  **Re-referencing:** All electrodes in this study were symmetrically distributed. To minimize the impact of the reference electrode on the experimental data, the EEG data were re-referenced using the average signal from all electrodes.
**(4)**  **ICA for artifact removal:** ICA, based on the statistical properties of signals, effectively separates overlapping EEG signals, removing noise and artifacts from the original EEG data to enhance data quality.

#### 2.2.2. Data Annotation

After preprocessing the EEG signals, the data were segmented and annotated for subsequent spatial cognition detecting experiments. The EEG signals were segmented using the disappearance time of each observed vehicle as the starting time step. A 1.5 s segment, consisting of 192 time steps including the starting step, was treated as a single dataset. Next, relative distance labels (short, medium, long) were assigned to the EEG data based on the participants’ marked distance perceptions. Additionally, relative orientation labels (left, front, right) were assigned using the recorded positional data of the observed vehicles.

## 3. Methodology

As described above, our spatial cognition recognition experiment involved two classification tasks: a three-class classification of relative distance (short, medium, long) and a three-class classification of relative orientation (left, front, right). To address these tasks, we designed a novel neural network, DTFNet, to decode the EEG signals effectively. For comparison, we selected three traditional machine learning methods and three deep learning-based methods as baselines for the experiments.

### 3.1. EEG Feature Extraction

**Figure 4:**
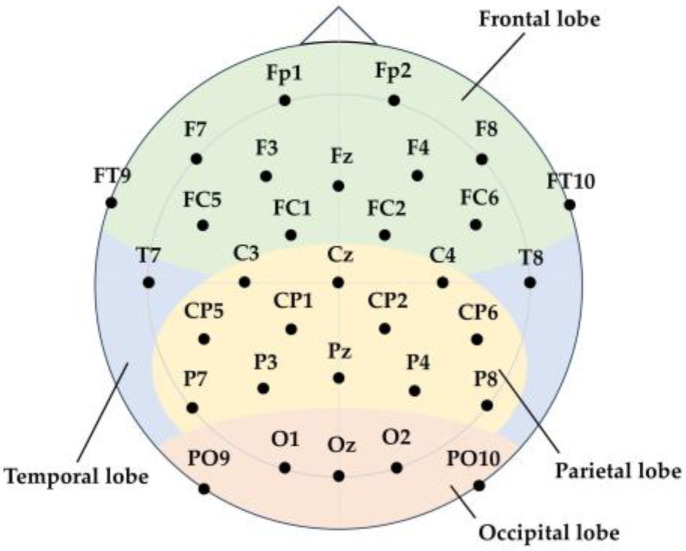
Electrode distribution of the EEG acquisition device.

Since traditional machine learning algorithms face challenges in directly processing high-dimensional data such as raw EEG signals [41], we performed feature extraction on the EEG signals to reduce the dimensionality of the data. As shown in Figure 4, the EEG electrodes were evenly distributed across the brain, with each electrode corresponding to a specific brain region. Based on their positions, the electrodes were categorized into four regions: frontal, parietal, occipital, and temporal lobes [42]. The specific electrodes corresponding to each region are listed in Table 1.

EEG signals can be divided into frequency bands, including Theta waves (3–8 Hz), Alpha waves (8–12 Hz), Beta waves (12–27 Hz), and Gamma waves (27 Hz and above) [43]. Among these, Alpha waves have been shown to correlate with spatial cognition and spatial attention abilities [29]. Therefore, we calculated the power spectral density (PSD) of the Alpha frequency band for the four brain regions to extract features relevant to the driver’s spatial cognition recognition task. This resulted in the extraction of four features for each set of EEG signals [44].

We took 96 or 128 time steps as a sampling window and calculated the power spectral density of each channel for the data in each sampling window. For each partition, the average power spectral density of all channels within the partition was used as a feature. Regarding a discrete-time signal that has a length of *N*, the approach for computing its PSD is as follows:

(2) $${\hat{S}}_{x}(f)=\frac{1}{N}{\left|X_{N}(f)\right|}^{2}$$

Here, $X_{N}(f)$ refers to the discrete Fourier transform (DFT) of x[n].

In this research, in order to mitigate the influence of spectral leakage, each segment of the signal was dealt with by applying a Hann window prior to calculating the PSD. The Hann window function is defined in this way:

(3) $$w(n)=0.5(1-\cos(\frac{2\pi n}{N-1})),\;n=0,1,\ldots ,N-1$$

The overall power within a particular frequency band can be acquired by adding up the PSD values across the intended frequency range, which is expressed as

(4) $$P_{band}=\sum_{f_{1}}^{f_{2}}{\hat{S}}_{x}(f)\Delta f$$

In the above formula, $f_{1}$ and $f_{2}$ stand for the lower and upper frequency limits, respectively, and Δf represents the frequency resolution.

### 3.2. Traditional Machine Learning Algorithms

Using the extracted features as input, we employed three traditional machine learning algorithms to perform the driver’s spatial cognition detecting task: K-Nearest Neighbors (KNNs) [45], Support Vector Machine (SVM) [46], and Random Forest (RF) [47]. The parameters of these algorithms are detailed in Table 2.

### 3.3. Deep Learning Algorithms

With the advancement of deep learning algorithms, many studies on EEG signal decoding have shifted away from manual feature extraction. Instead, they focus on designing neural networks to automatically extract features and perform decoding. In this study, three neural networks specifically designed for EEG signal processing were employed for feature extraction and classification: MLP [48], EEGNet [49], and ConvNet [50]. Additionally, we propose a novel neural network based on dual-time feature fusion, which achieves the best decoding performance. The following sections provide a detailed introduction to these neural networks.

#### 3.3.1. MLP-Based Model

This study constructs two spatial cognition decoding models based on the Multi-Layer Perceptron (MLP). The primary distinction between the two models lies in whether the EEG signals in the input dataset have undergone frequency domain feature extraction. Both models share a similar basic architecture, consisting of a fully connected network with four hidden layers. To prevent overfitting and enhance generalization, the dropout technique is applied for regularization. Assume that the input of the model is $x\in R^{n}$ and the output of the model is $y\in R^{m}$. Then, the MLP model can be represented as

(5) y=f(Wx+b)

where $W\in R^{n\times m}$ is the weight matrix, b is the bias, and f is the nonlinear activation function.

The input size of the raw EEG signals is either 32 × 96 or 32 × 128, representing the number of channels and time steps, respectively. For manually extracted features, the input size is 1 × 4, corresponding to the four features derived from the Alpha band power spectral density of different brain regions. These two MLP models were used to compare the impact of manual feature extraction versus direct feature extraction using deep learning models on the final classification performance of EEG signals. This comparison highlights the effectiveness of automated feature extraction in decoding spatial cognition from EEG data.

#### 3.3.2. EEGNet

The spatial cognition decoding from the EEG task introduced in this paper is a novel task. As no neural network models specifically designed for this task currently exist, we have chosen general EEG signal decoding models for evaluation.

The first model selected is EEGNet, a compact convolutional neural network tailored for EEG signal decoding tasks. EEGNet features a unique convolutional structure, incorporating depthwise convolution, separable convolution, and pointwise convolution, enabling it to efficiently extract EEG signal features. This model combines high efficiency—characterized by a small number of parameters, low computational complexity, and suitability for real-time applications—with strong generalization capabilities, performing effectively across diverse brain–computer interface paradigms. EEGNet is considered a baseline in many EEG signal decoding studies. The specific structure and parameters of EEGNet utilized in this study are presented in Table 3.

#### 3.3.3. ConvNet

ConvNet, proposed by R. Schirrmeister et al. [50], is a neural network designed for EEG signal classification tasks. The authors introduced two architectures, ShallowConvNet and DeepConvNet, which achieved state-of-the-art performance in distinguishing pathological EEG signals from normal ones. These architectures have since been established as general-purpose neural networks for EEG signal decoding.

In this study, both ShallowConvNet and DeepConvNet were employed to perform the spatial cognition decoding task. The detailed parameters of these two networks are presented in Table 4 and Table 5, respectively.

#### 3.3.4. DTFNet

In this study, we developed a novel neural network, Dual-Time-Feature Net (DTFNet), to address the spatial cognition decoding task. The model is divided into two main modules: a temporal processing module and a spatial processing module. The structure and components of these two modules are introduced below.

**(1)**  **Temporal Processing Module:** The temporal processing module comprises a time-dimension convolutional module and a GRU module. Considering that convolutional modules often struggle to capture long-term dependencies in EEG signals, the GRU module [51] is incorporated to extract fine-grained temporal features for each EEG channel. These features are then fused with the coarse-grained temporal features extracted by the convolutional module, enabling the model to effectively handle both short-term and long-term temporal dependencies.
**(2)**  **Spatial Processing Module:** The spatial processing module is designed to address the challenge of determining which of the 32 EEG channels contain the most relevant information. To achieve this, we utilize the Squeeze-and-Excitation (SE) module [52], which computes channel attention to distinguish the contributions of different channels. Afterward, the features from all channels are fused using a spatial dimension convolutional module to generate the final spatiotemporal features. These features are passed to a fully connected layer to produce the final prediction results.

**Figure 5:**
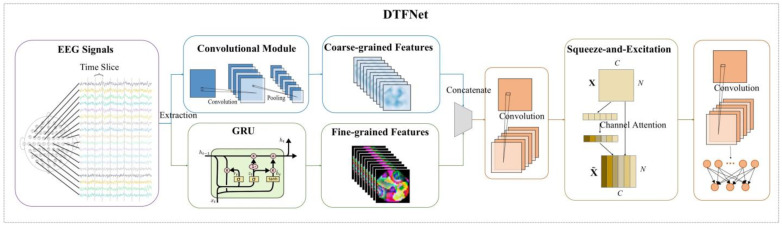
The structure of the DTFNet.

By integrating temporal features at two scales and leveraging spatial attention mechanisms, DTFNet demonstrates superior performance compared to general EEG decoding models in spatial cognition decoding tasks, making it a robust and efficient solution for decoding drivers’ spatial cognition from EEG signals. The architecture of DTFNet is illustrated in Figure 5, and the parameters of the model are detailed in Table 6, offering a clear understanding of the network’s design and implementation.

For a given input $x\in R^{C\times T}$, it is first processed through two temporal feature extraction modules to obtain coarse-grained temporal features and fine-grained temporal features.

(6) $${\mathrm{Feature}}_{coarse}=\mathrm{ELU}(\mathrm{Avg}(\mathrm{Conv}2d\_1(x)))$$

(7) $${\mathrm{Feature}}_{fine}=\mathrm{ELU}(\mathrm{GRU}(x))$$

Next, the coarse-grained and fine-grained temporal features are concatenated and passed through a convolutional layer to perform feature fusion. This process integrates the complementary information from both feature types, resulting in a unified representation of the temporal features.

(8) $${\mathrm{Feature}}_{temporal}=\mathrm{ELU}(\mathrm{Conv}2d\_2(\mathrm{Concat}({\mathrm{Feature}}_{coarse},{\mathrm{Feature}}_{fine})))$$

To effectively fuse the features of the EEG signals along the spatial dimension, we employ the SE module. This module starts by averaging the features of each channel through an average pooling layer, generating a global descriptor for each channel. These descriptors are then passed through two fully connected layers with an activation function in between, producing channel-specific weights. Finally, these weights are applied to the original features via element-wise multiplication, amplifying the contributions of relevant channels and reducing the influence of less informative ones.

(9) $${\mathrm{Weight}}_{channel}=\mathrm{Sigmoid}\;(\mathrm{FC}\;(\mathrm{ReLU}\;(\mathrm{FC}\;(\mathrm{Avg}\;({\mathrm{Feature}}_{temporal})))))$$

(10) $${\mathrm{Feature}}_{channel}={\mathrm{Weight}}_{channel}⊙{\mathrm{Feature}}_{temporal}$$

After the SE module, a convolutional layer is applied to fuse the features across the channel scale, resulting in the final spatiotemporal feature representation. Finally, these features are passed through a fully connected layer to generate the prediction results.

(11) $${\mathrm{Feature}}_{spatiotemporal}=\mathrm{ELU}(\mathrm{Conv}2d\_3({\mathrm{Feature}}_{channel}))$$

(12) $$\mathrm{Output}=\mathrm{FC}({\mathrm{Feature}}_{spatiotemporal})$$

## 4. Experimental Results

This section presents the classification results for the spatial cognition decoding task. For traditional machine learning models and one of the MLP models, the inputs are EEG signals that have undergone feature extraction. In contrast, the inputs for the other deep learning models consist of EEG signals that have been preprocessed but not feature-extracted. We employed the five-fold cross-validation method to rigorously evaluate the classification performance of all models in this study. Five-fold cross-validation divides the dataset into five subsets. For each iteration, one subset is used as the test set, while the remaining four subsets are used to train the model. This approach provides a robust evaluation of model performance by ensuring that all data are used for both training and testing across the five iterations.

The models were implemented using the PyTorch (Version 1.8.1) framework and the Scikit-learn library. All training was conducted on an NVIDIA GeForce GTX TITAN GPU. A learning rate of 0.0001 was applied to all models, and each model was trained for 500 epochs. To evaluate the classification performance, we adopted four standard metrics: accuracy, precision, recall, and F1 ccore. These metrics provide a comprehensive assessment of the models’ effectiveness in the spatial cognition decoding task.

### 4.1. Relative Orientation Classification Task

We first conducted the relative orientation classification task, and the experimental results are presented in Table 7. This task evaluates the spatial cognition of drivers in the orientation dimension. Successfully decoding drivers’ relative orientation cognition of a target from EEG signals can provide valuable insights for autonomous driving systems. By accurately understanding the orientation of the target that the driver is focusing on, the driving system can make informed and reasonable decisions that align with the driver’s intentions. This helps to prevent situations where the autonomous driving system’s actions contradict the driver’s expectations, thereby enhancing system reliability and driver satisfaction.

The relative orientation decoding task was simplified into a three-class classification task involving the categories left, front, and right, resulting in a chance-level probability of 33.33% for the classification task. For this task, a time window size of 96 was selected for the EEG signals, meaning that the input size is $x\in R^{32\times 96}$. The rationale for selecting this specific time window size will be discussed in detail in the Section 4.3.

From the results presented in Table 7, it can be observed that, with the exception of the feature-based MLP classifier, the classification performance of all other models surpasses the chance probability. We attribute the failure of the feature-based MLP classifier to the low dimensionality of the extracted features, which likely hindered effective training of the neural network. When comparing traditional machine learning algorithms with deep learning algorithms, it is evident that deep learning approaches achieve superior classification performance. This difference can be explained by two factors:

**(1)**  **Limited Relative Spatial Cognition Information in Extracted Features:** The features obtained through manual extraction contain relatively sparse information about relative orientation, which negatively impacts classification performance.
**(2)**  **Superior Feature Extraction and Learning Capabilities of Deep Learning Models:** Deep learning algorithms demonstrate more robust capabilities in automatically extracting and learning relevant features directly from EEG signals, leading to improved classification outcomes.

Our proposed algorithm achieves the best classification performance, with an accuracy of 65.67%, precision of 65.90%, recall of 65.72%, and an F1 score of 65.61%. These results highlight the effectiveness of our approach for the relative orientation decoding task.

### 4.2. Relative Distance Classification Task

The relative distance decoding task, similar to the relative orientation decoding task, was also simplified into a three-class classification problem, involving the categories close, medium, and far. This task, however, is more challenging than the relative orientation decoding task because the perception of relative distance is inherently more subjective. Upon analyzing the collected data, we identified a certain degree of overlap between the actual distances corresponding to the perceived categories of close, medium, and far. To address this issue, we excluded data with cognitive ambiguity before proceeding with the decoding task. Successfully decoding individuals’ perception of relative distance from EEG signals can assist autonomous driving systems in better adjusting safe distances and improving driver comfort. For this task, a time window size of 128 was selected for the EEG signals; that is, the input is $x\in R^{32\times 128}$.

From the experimental results presented in Table 8, it can be observed that although decoding relative distance from EEG signals is inherently challenging, the classification accuracy of most models exceeds the chance probability, demonstrating the feasibility of decoding spatial cognition from EEG signals. Among the models, deep learning approaches consistently outperform traditional machine learning models, with the exception of the MLP model, which failed to achieve competitive classification performance. This highlights the superior capability of deep learning algorithms in feature extraction and learning from raw EEG signals. Our proposed model achieved the best classification performance, with an accuracy of 50.65%, precision of 50.68%, recall of 50.64%, and an F1 score of 49.91%. These results validate the effectiveness of our model for addressing the relative distance decoding task.

### 4.3. Further Discussion

To gain deeper insights into the experimental results, we decomposed the three-class classification problem into three binary classification problems. This approach allowed us to analyze the classification performance for each pair of categories individually, providing a more granular understanding of the challenges and strengths associated with decoding spatial cognition from EEG signals.

Furthermore, we explored the temporal dynamics of drivers’ spatial detection, examining how drivers’ spatial cognition evolves over time. This temporal analysis offers valuable insights into the relationship between EEG signals and drivers’ perception of relative distances, which may further inform the development of adaptive and responsive autonomous driving systems.

We also provided an in-depth analysis of the challenges this system may face in real-world applications. Additionally, we proposed potential solutions to these challenges and identified promising avenues for future research.

#### 4.3.1. Binary Classification Results

To further analyze the two spatial cognition decoding tasks, we simplified the three-class classification problems into multiple binary classification tasks. For instance, in the relative orientation classification problem, the task was divided into the following binary classification problems: Left vs. Front, Left vs. Right, and Front vs. Right. This division allows for a more detailed assessment of the performance of various methods on specific pairwise comparisons, offering deeper insights into their strengths and weaknesses. The classification accuracies of different methods for these binary classification problems are presented below.

**(1)**  **Binary classification results of relative orientation**

**Figure 6:**
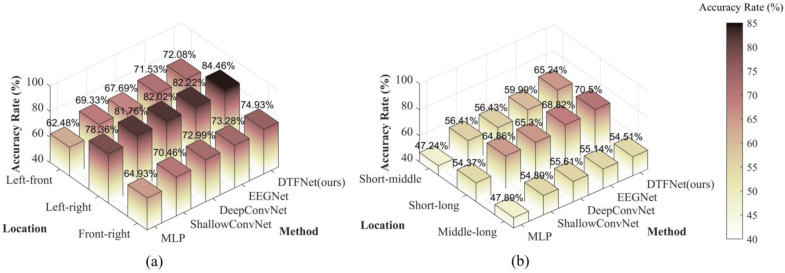
Binary classification results. (**a**) Relative orientation and (**b**) relative distance.

The binary classification results for the relative orientation decoding task are displayed on Figure 6a. The results indicate that all models achieved classification accuracies exceeding the chance probability of 50%. From the figure, it is evident that the left-right binary classification task yields the best performance. This aligns with our hypothesis, as the cognitive distinction between left and right orientations is significantly greater than that for the other two binary classification tasks. For the left-front and front-right binary classification tasks, the classification performances are nearly identical, reflecting the symmetrical experimental scenarios used in these cases.

In summary, the results demonstrate that drivers’ spatial cognition of relative orientation can be effectively decoded from EEG signals. Moreover, the findings indicate that drivers’ cognitive processing of left and right orientations does not exhibit a strong inherent bias. Finally, while the performance differences among several deep learning models are minimal across the binary classification tasks, our proposed model consistently achieves the best classification performance, highlighting its effectiveness in detecting drivers’ spatial cognition.

**(2)**  **Binary classification results of relative distance**

The binary classification results for the relative distance decoding task are displayed in Figure 6b. As shown, the decoding performance for relative distance is inferior to that for relative orientation, consistent with the findings of the three-class classification study discussed earlier. Similar to the binary classification results for relative orientation, the short-long group achieves the best classification performance among the relative distance tasks. However, unlike relative orientation, relative distance is not a symmetrical scenario, and the decoding performance for the short-medium and medium-long groups differs significantly. The figure shows that the decoding performance for the short-medium group is better than that for the medium-long group. This result aligns with the cognitive patterns of human drivers. During driving, drivers tend to focus more on targets that are closer to them, as closer targets pose a higher potential danger. Conversely, targets at medium and long distances are less likely to pose an immediate threat, resulting in less pronounced differences in EEG signals for these group.

#### 4.3.2. Temporal Dynamics of Drivers’ Spatial Cognition

**Figure 7:**
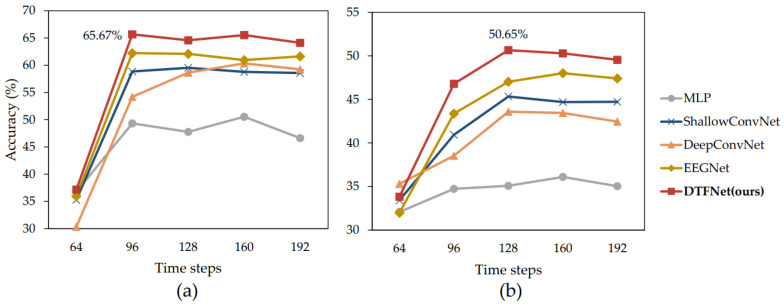
Detecting results with different time window sizes. (**a**) Relative Orientation and (**b**) relative Distance.

We analyzed the temporal dynamics of drivers’ spatial cognition using EEG signals. The sampling frequency of our EEG acquisition instrument is 128 Hz, meaning that 128 time steps correspond to 1 s in real time. EEG decoding began with 64 time steps and was repeated at intervals of 32 additional time steps, up to 192 time steps. The decoding results for relative orientation and relative distance are shown in Figure 7a,b, respectively. From the figures, it is evident that when the time step length is 64, it is difficult to extract effective spatial cognition information from EEG signals. This is likely because spatial cognition requires a certain amount of processing time. After visual stimuli are received by the retina, they must be transmitted to the visual processing cortex, where spatial cognition is formed [53]. And by comparing the results for the two tasks, the following observations can be made:

**(1)**  **Relative Orientation Decoding Task**: The best decoding performance is achieved when the EEG signal time step length is 96. Increasing the time window size beyond 96 does not significantly enhance classification performance, suggesting that orientation cognition stabilizes within this time frame.
**(2)**  **Relative Distance Decoding Task:** The optimal decoding performance is achieved at a time step length of 128, indicating that the perception of relative distance occurs slightly later than that of orientation.

Additionally, across all time step lengths, our proposed model consistently outperforms the other models in classification accuracy. This demonstrates the robustness and effectiveness of our model for decoding drivers’ spatial cognition in both relative orientation and relative distance tasks.

#### 4.3.3. Application Analysis and Limitations

Although our designed human-centric spatial cognition detecting system can detect the spatial perception of drivers, enabling the driving system to establish a more comfortable following distance and maintain the vehicle within the safe range perceived by the drivers based on the position of the target they are focusing on, there is still a gap before it can be applied in real-world scenarios, and several challenges remain to be addressed.

The first challenge lies in the significant gap between the simulated scenarios used in our study and real-world driving conditions. In real driving scenarios, drivers encounter multiple targets moving continuously, and the resulting neural signals may differ from those observed in our simulated environment. However, even in real-world scenarios, drivers still engage in relative orientation and distance cognition of targets. Therefore, we believe that the EEG signals should not differ substantially. In the future, we plan to further refine our simulated scenarios to make them more closely resemble real-world conditions and conduct experiments in real driving environments to collect and analyze data for further research.

Another challenge is the real-time decoding of EEG signals. Unlike the current experiments, where EEG signals are segmented and then decoded, practical applications require real-time analysis. Real-time EEG decoding is an active area of research in the field of brain–computer interfaces, but it is not the primary focus of this study. In the future, we could explore integrating a real-time EEG decoding system into our framework. This would enable continuous analysis of EEG signals, allowing us to provide ongoing outputs of the driver’s spatial perception, thereby contributing to the decision-making process of the driving system.

The driver’s state can also affect the detecting performance. In real-world scenarios, drivers may experience fatigue during driving. In cases of mild fatigue, the detection of spatial cognition—being a fundamental cognitive ability—may not be significantly affected. However, in cases of moderate to severe fatigue, further processing of the EEG signals will be necessary before detection. We consider using preprocessing algorithms, such as wavelet transform, or integrating existing driver fatigue detection algorithms to design a denoising module based on deep learning. Additionally, during actual driving, the driver’s head movements and driving actions can introduce more artifacts, degrading the quality of the EEG signals. We plan to apply ICA or deep learning algorithms to remove these artifacts and improve the decoding algorithm to enhance performance on low-quality EEG signals.

In the future, we will conduct further research on the three challenges mentioned above, improving the existing simulation environment to make it more closely resemble real-world scenarios. At the same time, we will strive to enhance the robustness and accuracy of the driver’s spatial cognition detecting system.

## 5. Conclusions

In response to the limitations of existing autonomous driving systems, particularly their limited ability to mimic human-like decision-making, this paper proposes a human-centric spatial cognition detection system based on drivers’ signals for autonomous driving. The system decodes two critical dimensions of drivers’ spatial cognition—relative distance and relative orientation—from EEG signals. Our findings demonstrate that spatial cognition can be effectively extracted from EEG data. To achieve this, we propose a novel EEG signal decoding neural network, DTFNet, which integrates temporal features at multiple scales. The proposed network achieves superior performance compared to other general-purpose EEG decoding models in spatial cognition tasks. Binary classification experiments reveal that drivers focus more on closer targets, while exhibiting no significant directional preference. Furthermore, our exploration of the temporal dynamics of drivers’ spatial cognition indicates that drivers perceive relative orientation slightly earlier than relative distance.

In the future, our work will be carried out in two main directions. On the one hand, we will enhance the complexity of experimental scenarios to better simulate real-world driving conditions and collect a larger and more diverse dataset of drivers’ spatial cognition to improve the system’s robustness and generalizability. On the other hand, we will develop EEG signal decoding algorithms specifically tailored to spatial cognition tasks, focusing on improving the accuracy and effectiveness of decoding drivers’ spatial cognition and addressing the unique challenges posed by this application.

## Figures and Tables

**Table 1:** Electrode partition.

Region	Electrode
Frontal lobe	‘Fp1’, ‘Fp2’, ‘Fz’, ‘F3’, ‘F4’, ‘F7’, ‘F8’, ‘FC1’, ‘FC2’, ‘FC5’, ‘FC6’, ‘FT9’, ‘FT10’
Parietal lobe	‘Cz’, ‘C3’, ‘C4’, ‘CP1’, ‘CP2’, ‘CP5’, ‘CP6’, ‘Pz’, ‘P3’, ‘P4’, ‘P7’, ‘P8’
Occipital lobe	‘Oz’, ‘O1’, ‘O2’, ‘PO9’, ‘PO10’
Temporal lobe	‘T7’, ‘T8’

**Table 2:** Parameters of the three methods.

Method	Parameters
K-Nearest Neighbors (KNNs)	K = 30
Support Vector Machine (SVM)	Kernel = ‘poly’, C = 10, random_state = 42
Random Forest (RF)	Trees = 1000, Max depth = None, random_state = 42

**Table 3:** The model structure and parameters of EEGNet.

Layer	Parameters	Activation
Conv2D	input = 1, output = 16, kernel_size = (1, 64), padding = ‘same’	-
BatchNorm	8	-
Conv2D	input = 16, output = 2 × 16, kernel_size = (32, 1), groups = 8, max_norm = 1	-
BatchNorm	16	ELU
AveragePool2D	kernel_size = (1, 4)	-
Dropout	0.25	-
Conv2D	input = 2 × 16, output = 32, kernel_size = (1, 16), groups = 16, padding = ‘same’	-
Conv2D	input = 32, output = 32, kernel_size = 1	-
BatchNorm	32	ELU
AveragePool2D	kernel_size = (1, 8)	-
Dropout	0.25	-
Flatten	-	-
FC	input = 64, output = 3, max_norm = 0.25	Softmax

**Table 4:** The model structure and parameters of ShallowConvNet.

Layer	Parameters	Activation
Conv2D	input = 1, output = 25, kernel_size = (1, 25)	-
Conv2D	input = 25, output = 25, kernel_size = (32, 1)	-
BatchNorm	25	ELU
AveragePool2D	kernel_size= (1, 15), stride = 5	-
Dropout	0.4	-
Flatten	-	-
FC	input = 450, output = 3	Softmax

**Table 5:** The model structure and parameters of DeepConvNet.

Layer	Parameters	Activation
Conv2D	input = 1, output = 25, kernel_size = (1, 5), stride = (1, 2)	-
Conv2D	input = 25, output = 25, kernel_size = (32, 1),	-
BatchNorm	25	ELU
MaxPool2D	kernel_size = (1, 3), stride = 1	-
Dropout	0.4	-
Conv2D	input = 25, output = 50, kernel_size = (1, 5), stride = (1, 2)	-
BatchNorm	50	ELU
MaxPool2D	kernel_size = (1, 3), stride = 1	-
Dropout	0.4	-
Conv2D	input = 50, output = 100, kernel_size = (1, 5), stride = (1, 2)	-
BatchNorm	100	ELU
MaxPool2D	kernel_size = (1, 3), stride = 1	-
Dropout	0.4	-
Flatten	-	-
FC	input = 200, output = 3	Softmax

**Table 6:** The parameters of the DTFNet.

Layer	Parameters	Activation
GRU	input = 1, hidden_size = 64, num_layers = 1	ELU
Conv2D_1	Input = 1, output = 16, kernel_size = (1, 64), padding = ‘same’	-
BatchNorm	16	ELU
AveragePool2D	(1, 2)	-
Dropout	0.5	-
Conv2D_2	input = 1, output = 16, kernel_size = (1, 128)	-
BatchNorm	16	ELU
Dropout	0.5	-
AveragePool2D	(None, 1)	-
Flatten	-	-
Dense	input = 32, output = 16	ReLU
Dense	input = 16, output = 32	Sigmoid
Dropout	0.5	-
Conv2D_3	input = 1, output = 16, kernel_size = (32, 1)	-
BatchNorm	16	ELU
Flatten	-	-
FC	input = 256, output = 3	Softmax

**Table 7:** The results of the relative orientation classification task.

Models	Accuracy (%)	Precision (%)	Recall (%)	F1 Score (%)
KNN	37.30	37.62	37.30	37.01
SVM	38.22	40.31	38.22	31.46
RF	34.63	35.23	34.62	34.59
MLP_feature	32.78	32.70	32.79	32.18
MLP_raw	49.32	48.41	49.30	46.92
EEGNet	62.63	62.43	62.25	62.03
ShallowConvNet	58.85	59.18	58.90	58.78
DeepConvNet	54.19	55.75	54.53	54.00
**DTFNet (ours)**	**65.67**	**65.9** **0**	**65.72**	**65.61**

**Table 8:** The results of the relative distance classification task.

Models	Accuracy (%)	Precision (%)	Recall (%)	F1 Score (%)
KNN	40.10	41.55	40.10	41.07
SVM	36.29	41.52	36.29	29.55
RF	39.99	40.87	39.99	39.91
MLP_feature	33.89	34.27	33.89	33.40
MLP_raw	35.09	35.02	35.01	34.90
EEGNet	47.02	46.68	46.98	45.99
ShallowConvNet	45.33	45.31	45.26	45.04
DeepConvNet	43.59	48.80	43.81	40.99
**DTFNet (ours)**	**50.65**	**50.68**	**50.64**	**49.91**

## Data Availability

The data presented in this study are available on request from the corresponding author.
